# Evidence on factors influencing contraceptive use and sexual behavior in South Africa: A systematic scoping review protocol

**DOI:** 10.1097/MD.0000000000013774

**Published:** 2018-12-28

**Authors:** Mbuzeleni Hlongwa, Tivani Mashamba-Thompson, Khumbulani Hlongwana

**Affiliations:** Discipline of Public Health Medicine, School of Nursing and Public Health, University of KwaZulu-Natal, Durban, South Africa.

**Keywords:** abortions, contraceptive use, HIV/AIDS, pregnancy, sexual behavior, South Africa

## Abstract

**Background:**

Contraceptive use and sexual health behavior remain a prominent public health concern in South Africa. Despite many government interventions, unplanned pregnancies, number of abortions, and maternal mortality remain relatively high. Due to high pregnancy levels and the stigma associated with termination of pregnancy, more women turn to unsafe and illegal abortions despite the risks involved. Risky sexual behavior pose a serious risk of contracting HIV/AIDS. The main objective of this study is to map evidence on factors influencing contraceptive use and sexual behavior in South Africa.

**Methods:**

We will conduct a scoping review guided by framework by Arksey and O’Malley. This study will search for eligible literature from peer-reviewed articles and grey literature. Databases such as PubMed/MEDLINE, American Doctoral Dissertations via EBSCO host, Union Catalogue of Theses and Dissertations (UCTD) and SA ePublications via SABINET Online and World Cat Dissertations, Theses via OCLC, and Google Scholar will be searched. Websites such as the World Health Organization (WHO) and governmental websites and statistics institutions will be explored for policies and guidelines on contraceptive use and sexual behavior. The review will be conducted on studies that were published from January 1990 to 2018. The PCC framework will be employed in this study to determine the eligibility of research question. The PRISMA chart will be utilized to report the screening of results. The MMAT Tool version 11 will be used to determine the quality of the included primary studies.

**Results:**

We anticipate finding a considerable number of published articles presenting evidence on contraceptive use and sexual health behavior in South Africa. Findings of this scoping review will be disseminated electronically, in print, and through peer presentation, conferences, and congresses.

## 1 Introduction

Contraceptive use, sexual health behavior, and HIV/AIDS education remain prominent in demographic and health literature because of their several health benefits to women and families, such as preventing unplanned pregnancies, reducing maternal mortality, and the risk of HIV infection, particularly in the African region.^[1]^ The sub-Saharan African region experiences more than 14 million unplanned pregnancies each year, with almost half of these pregnancies happening among women aged 15 to 24 years.^[2]^ More than 13% of these pregnancies end in abortions and 16% in miscarriages.^[1]^ As a result of maternal-related complications, 1 in 26 women of reproductive age die in Africa, compared with 1 in 9400 in Europe.^[3]^ In 2003, the prevalence of unplanned pregnancy and contraceptive use in South Africa were 47% and 62%, respectively. In 2013 alone, the teenage and adolescents’ unplanned pregnancies exceeded 99,000 pregnancies in South Africa.

Maternal factors such as pregnancy and childbirth complications are the leading cause of death among 15 to 19-year-old girls worldwide. Almost all of the deaths affecting the low and middle-income countries account for 99% of maternal mortality among women aged 15 to 49 years, globally.^[4]^ In response to this reproductive health challenge, the South African government has adopted the Sustainable Development Goals (SDGs) commitments, which aim to ensure universal access to sexual and reproductive health for all women by 2030. Some of the main objectives of the department of health in South Africa is to reduce the maternal mortality in facility ratio to 100 (or less) per 100,000 live births and increase the contraceptive use rate to 75% by 2020. In addition to the above strategic goals, this study will further contribute to Chapter 10 (Promoting Health) of the National Development Plan (NDP) vision 2030, which aims to significantly reduce the sexually transmitted infections (STIs) and the burden of HIV/AIDS. This study will further address Goal 3 of the NDP vision 2030, which aims to reduce maternal mortality rates in South Africa.

Despite the improved contraception uptake in South Africa, there remains high termination of pregnancy due to the high numbers of undesirable and unintended pregnancies, posing a serious concern for public health. The situation is exacerbated by a relatively high HIV/AIDS infection rate, particularly among adolescents. Due to such high pregnancy levels and the stigma associated with termination of pregnancy, more women turn to unsafe and illegal abortions negating the risks associated with such activities. The main objective of this study is to map evidence on factors influencing contraceptive use and sexual behavior in South Africa over a period spanning from 1990 to 2018.

## 2 Methodology

### 2.1 Systematic scoping review

This study protocol is registered under the following URL: https://nhrd.hst.org.za/Proposal/Details/43106 (registration no.: KZ_201809_013). This study will be conducted using a scoping review of published peer-reviewed and grey literature on the factors influencing contraceptive use and sexual behavior in South Africa. The framework by Arksey and O’Malley^[5]^ on scoping review will guide this study. The following stages are specified by the framework:

(1) Identifying the research question;
(2) Identifying relevant studies;
(3) Study selection;
(4) Charting the data;
(5) Collating, summarizing, and reporting the results.

This review will also include the quality appraisal of included primary studies, which was recommended by Levac et al^[6]^ for scoping review projects.

### 2.2 Identifying the research question

What are the factors that influence contraceptive use and sexual behavior in women of child-bearing age (15–49 years) in South Africa?

### 2.3 Eligibility of research question

The Population, Concept, and Context (PCC) framework has been employed in this study to determine the eligibility of research question as illustrated in Table 1.

**Table 1:**
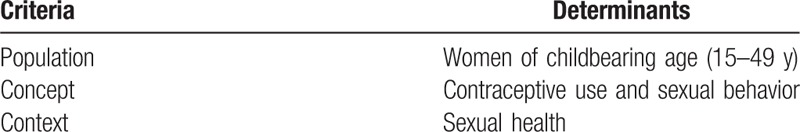
PCC framework.

### 2.4 Identifying relevant studies

This study will utilize evidence published by primary studies and grey literature, which have shown significant results using clear and strong methodologies in quantitative, qualitative, and mixed-method approaches. These studies will be obtained from the published peer-reviewed journals. The article searches will be inclusive of databases such as PubMed/MEDLINE, American Doctoral Dissertations via EBSCO host, Union Catalogue of Theses and Dissertations (UCTD) and SA ePublications via SABINET Online and World Cat Dissertations, Theses via OCLC, and Google Scholar. Publications by MRC and HSRC will also be reviewed. Websites such as the World Health Organization (WHO) and governmental websites and statistics institutions will be searched for policies and guidelines on contraceptive use and sexual behavior. The literature will be conducted on studies that were published from January 1990 to 2018. All study designs will be included. The citations from the selected studies will also be screened, and the relevant articles from the reference lists will be searched. The search key words will include contraceptive use, family planning, sexual behavior, HIV/AIDS, South Africa, pregnancy, abortions, maternal mortality. The Boolean terms such as “AND” and “OR” will be used to combine or concentrate keywords in an advanced search and this helps eliminate inappropriate articles while focusing results to the area of research interest. The Medical Subject Headings (MeSH) terms will be used for control, indexing, and description of article records. A pilot database search was conducted to determine the feasibility using the scoping review method to answer our research question (Table 2).

**Table 2:**
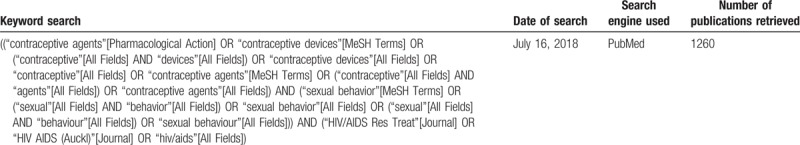
Pilot database search results.

### 2.5 Study selection

The eligibility criteria were designed to limit the study to focus only on the articles that address issues described in the research question: what are the factors influencing contraceptive use and sexual behavior in South Africa?

### 2.6 Inclusion criteria

The following principles will be used to determine the studies that meet the criteria:

(1) Studies that present evidence that was published between 1990 and 2018.
(2) Studies that present evidence that was published in South Africa.
(3) Studies that present evidence on women aged 15–49 years.
(4) Studies that present evidence on contraceptive use.
(5) Studies that present evidence on sexual behavior.
(6) Studies that present evidence on HIV/AIDS.

### 2.7 Exclusion criteria

Studies with the following characteristics will be excluded.

(1) Studies published before 1990
(2) Studies with no evidence on contraceptive use or sexual behavior

**Figure 1:**
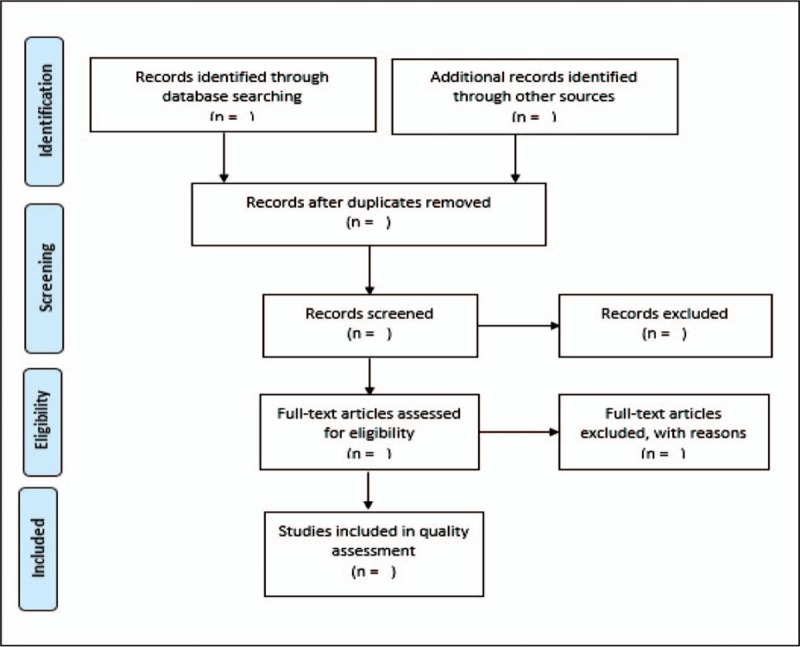
PRISMA flow chart demonstrating literature search and selection of studies.

Eligible articles from title screening will be exported to Endnote version 7 library, which will be created for the purpose of this scoping review. A comprehensive screening of study titles from the databases listed above will be conducted by the principal investigator. Although all studies meeting the inclusion criteria will be exported to Endnote, all studies will be checked for duplications and duplicate studies will be removed. This process will happen before the abstract screening is conducted. Following full article screening, we will conduct abstract screening. Two reviewers will screen abstracts as well as full articles. Each reviewer will work independently from each other and the screening will be guided by the above eligibility criteria. We will work closely with the University of KwaZulu-Natal library services during database searching and retrieval of articles. Studies that could not be retrieved from databases will be obtained by contacting authors. The Preferred Reporting Items for Systematic Reviews and Meta-Analysis (PRISMA) chart (Fig. 1) will be employed to report the screening of results.

### 2.8 Charting of data

The Data Charting table (Table 3) will be utilized as a guide to extract the background information that will be used for each study to be employed. Data charting form will be continually updated with the latest information and it will include the highlighting of the key aspects, which will be designed and piloted. Updating of the data charting form will be conducted continuously. NVivo version 12 will be used to seek out emerging themes from the included articles.

**Table 3:**
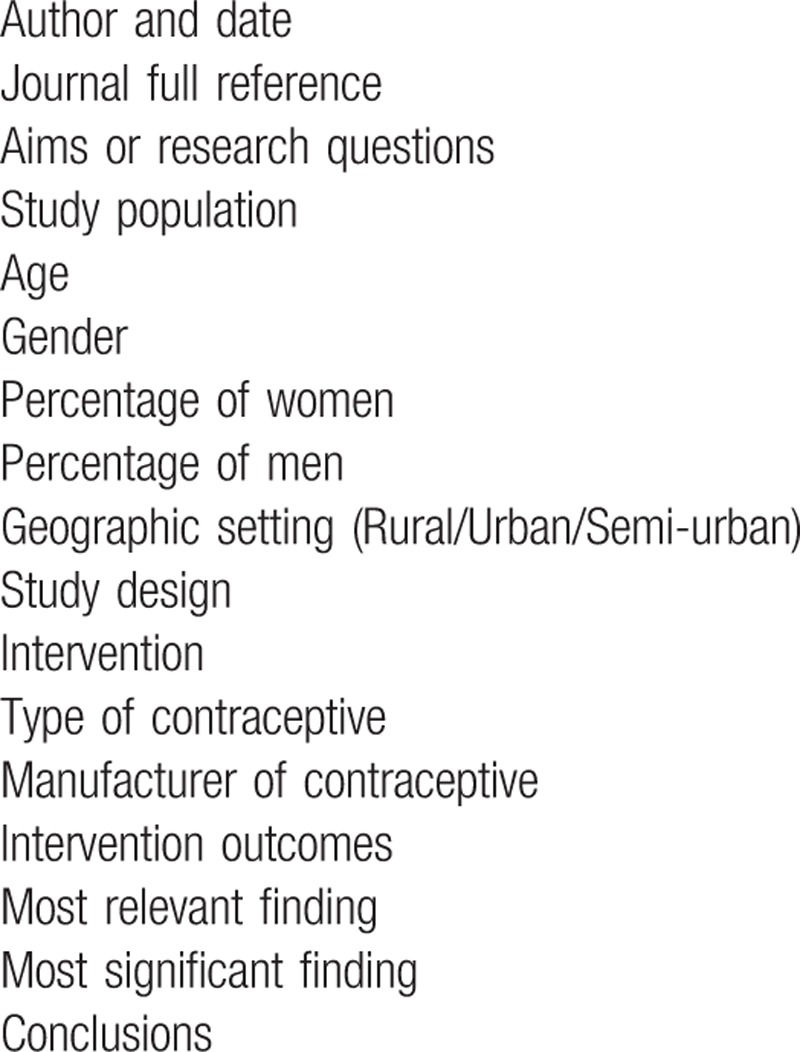
Data charting table form.

### 2.9 Collating, summarizing, and reporting the results

For coding and analyzing of data from the selected articles, content analysis of the extracted data will be conducted. The extent, nature, and distribution of the selected studies will be reviewed. To understand the context, content, population, geographical location, and research methods of the selected studies, a template with tables and charts mapping will be developed. This template with a table summarizing basic characteristics of all the selected studies will be designed in which reviewers will make comments or notes on the following headings: interventions; sample sizes; participants; research methods; evidence relating to effectiveness; economic aspects and gaps in the research.^[5]^ This consistent approach will help us make comparisons across intervention types; identify contradictory evidence regarding specific interventions; and identify research gaps.

### 2.10 Quality appraisal

To determine the quality of the selected studies, a Mixed Method Appraisal Tool (MMAT) version 2011 will be adopted and piloted by 2 people (principal investigator and coscreener). This tool was most appropriate for this study because it highlights the key aspects. Only primary studies will be assessed by the MMAT tool. This study will evaluate section 2, which focuses on randomized controlled studies, section 3 for nonrandomized controlled studies, and section 4, which measures descriptive statistics. Section 1 examines mixed methods studies for the qualitative component. This section will also be evaluated. The MMAT tool will be utilized to scrutinize the relevance of aim of study, adequacy and methodology, study design, data collection, study selection, data analysis, presentation of findings, author's discussions, and conclusions. The grey literature articles will be appraised using the Authority, Accuracy, Coverage, Objectivity, Date, Significance (AACODS) checklist form, which is designed to enable evaluation and critical appraisal of grey literature.^[7]^

The results from examination of the aspects indicated above will regulate quality of resultant article. Each study will be assigned an overall grade of high, moderate, or low risk of bias based on the assessment of the 6 sections indicated above. The following criteria will be followed: for qualitative and quantitative studies, the score will be a number of criteria met by each study divided by 4, with 25% indicating that at least 1 criterion was met by the study, while 100% indicates that all criteria were met.^[8]^ For the mixed methods studies, the score will be 25% when 1 criterion is met, 50% when 2 criteria are met for a domain, 75% when 3 criteria are met for a domain, and 100% when all criteria are met for all domains.^[8]^ Domains comprise of qualitative, quantitative, and mixed methods components.

## 3 Discussion

Most of the published systematic review articles mainly focus on adolescents and their choices of modern contraceptive use in sub-Saharan Africa.^[9–11]^ There are limited systematic reviews or scoping reviews conducted with a specific focus on contraceptive use and sexual behavior in general population and with a specific focus on South Africa. Universal contraception and improved sexual behavior has become a key priority for the South African government. The NDP and SDGs strategic plans specifically stress the importance of improving universal access to family planning services and educating the population on sexual behavior and HIV/AIDS.^[12]^ There is also a need to conduct more systematic reviews on contraceptive use and sexual behavior particularly among key populations, such as men who have sex with other men (MSM), sex workers, truck drivers, prisoners, and miners. In addition, there is very little sex or contraceptive education from parents, health care providers, or elsewhere in South Africa.

This systematic scoping review focuses on contraceptive use and sexual behavior in South Africa. It includes all studies published between the years 1990 and 2018 because studies published before 1990 are unlikely to reflect the key aspects and changes pertaining to modern contraceptive use and sexual health behavior. More studies were conducted after 1990 after many interventions were implemented to address these public health challenges in the era of HIV/AIDS.

We hope that the results of this scoping review will contribute to literature and policy guidelines on contraceptive use and sexual behavior of population aged 15 years and older, with a specific focus on HIV/AIDS in South Africa and other high HIV-pandemic countries. Findings of this scoping review will be disseminated electronically, in print, and through peer presentation, conferences, and congresses.

## Acknowledgment

The authors would like to thank the School of Nursing and Public Health, University of KwaZulu-Natal, Durban, South Africa.

## Author contributions

MH conceptualized, designed the study, and prepared the initial draft of the study under the supervision of KH and TPM-T. Both KH and TPM-T assisted with the manuscript preparation. All the authors reviewed the draft and approved the final version of the manuscript.

**Conceptualization:** Mbuzeleni Hlongwa, Khumbulani Hlongwana.

**Data curation:** Tivani Mashamba-Thompson, Khumbulani Hlongwana.

**Formal analysis:** Mbuzeleni Hlongwa, Tivani Mashamba-Thompson, Khumbulani Hlongwana.

**Investigation:** Mbuzeleni Hlongwa.

**Methodology:** Mbuzeleni Hlongwa, Tivani Mashamba-Thompson, Khumbulani Hlongwana.

**Project administration:** Mbuzeleni Hlongwa.

**Resources:** Khumbulani Hlongwana.

**Supervision:** Tivani Mashamba-Thompson, Khumbulani Hlongwana.

**Validation:** Mbuzeleni Hlongwa.

**Visualization:** Mbuzeleni Hlongwa, Khumbulani Hlongwana.

**Writing – original draft:** Mbuzeleni Hlongwa.

**Writing – review & editing:** Mbuzeleni Hlongwa, Tivani Mashamba-Thompson, Khumbulani Hlongwana.

Mbuzeleni Hlongwa orcid: 0000-0002-5352-5658.
